# Residual disease subtyping predicts survival and guides adjuvant immunotherapy in esophageal squamous cell carcinoma after neoadjuvant chemoimmunotherapy

**DOI:** 10.3389/fimmu.2026.1742272

**Published:** 2026-03-18

**Authors:** Huiya Wang, Haiyan Sun, Shuai Yi, Yingying Jin, Yao Lu, Ran Zuo, Yinli Yang, Ziyi Dong, Yaoyang Guo, Zhanyu Pan, Zhansheng Jiang, Xiaofeng Duan

**Affiliations:** 1 Department of Integrated Chinese and Western Medicine, Tianjin Medical University Cancer Institute & Hospital, National Clinical Research Center for Cancer, Tianjin’s Clinical Research Center for Cancer, Tianjin Key Laboratory of Digestive Cancer, Tianjin, China; 2 Department of Radiation Oncology, People's Hospital Of Huantai County, Zibo, Shandong, China; 3 Department of Oncology, The First Affiliated Hospital of Zhengzhou University, Zhengzhou, Henan, China; 4 Department of Minimally Invasive Esophageal Surgery, Tianjin Medical University Cancer Institute & Hospital, National Clinical Research Center for Cancer, Tianjin’s Clinical Research Center for Cancer, Tianjin Key Laboratory of Digestive Cancer, Tianjin, China

**Keywords:** adjuvant immunotherapy, esophageal squamous cell carcinoma, neoadjuvant chemoimmunotherapy, pathological complete response, prognostic nomogram, residual disease subtyping

## Abstract

**Background:**

The majority of locally advanced esophageal squamous cell carcinoma (ESCC) patients do not achieve a pathological complete response (NPCR) after neoadjuvant chemoimmunotherapy (NCIT), and their prognosis exhibits significant heterogeneity. This study aimed to establish a pathological subtyping system for NPCR patients to guide precision adjuvant therapy.

**Methods:**

We conducted a retrospective analysis of 243 patients with locally advanced ESCC who underwent NCIT followed by esophagectomy. NPCR patients were categorized into three pathological subtypes based on the anatomical sites of residual disease: T+N+ (residual tumor in both primary site and lymph nodes), T+N0 (residual tumor confined to primary site only), and T0N+ (residual disease in lymph nodes only). Survival outcomes were compared, and the efficacy of adjuvant immunotherapy was evaluated within each subtype. Prognostic nomograms for disease-free survival (DFS) and overall survival (OS) were constructed and validated.

**Results:**

Among 176 NPCR patients, the novel subtyping system achieved significant prognostic stratification. The T+N+ subtype demonstrated the poorest survival outcomes, while the T0N+ subtype did not significantly differ from the PCR cohort. Notably, adjuvant immunotherapy provided significant survival benefits exclusively in the T+N0 subtype (DFS: HR = 3.45, 95% CI: 1.17-10.17, P = 0.025; OS: HR = 4.17, 95% CI: 1.07-16.23, P = 0.039), with no significant benefits observed in either the T+N+ or T0N+ subtypes. Based on these findings, we developed and internally validated prognostic nomograms integrating pathological subtype, ypTNM stage, and other key clinicopathological variables, which demonstrated good predictive accuracy (C-index >0.75) for individualized risk assessment.

**Conclusion:**

We propose a novel and practical pathological subtyping framework for NPCR ESCC patients that effectively resolves prognostic heterogeneity and identifies, the T+N0 subtype as the primary beneficiary of adjuvant immunotherapy. The developed nomograms provide a user-friendly tool to facilitate personalized postoperative management and adjuvant therapy decisions.

## 1 Introduction

The management of locally advanced esophageal squamous cell carcinoma (ESCC) has evolved from surgery alone to a standard strategy centered on perioperative comprehensive therapy (1, 2). Neoadjuvant chemoradiotherapy (NCRT) followed by esophagectomy has been widely demonstrated to improve patient survival (3, 4). In recent years, with the widespread application of immune checkpoint inhibitors, the novel modality of neoadjuvant chemoimmunotherapy (NCIT) has shown encouraging rates of pathological complete response and promising short-term outcomes in both clinical trials and real-world studies, rapidly reshaping the treatment landscape for ESCC (5). With the advent of immune checkpoint inhibitors, the treatment landscape has been evolving rapidly. Programmed cell death protein 1 (PD-1), a receptor on activated T cells, transmits inhibitory signals upon binding to tumor-expressed PD-L1, leading to T-cell exhaustion and immune evasion. PD-1 inhibitors, as monoclonal antibodies, block this interaction to restore anti-tumor immunity (6).The integration of immunotherapy into neoadjuvant regimens has emerged as a promising approach. The phase II Keystone-001 trial evaluated neoadjuvant pembrolizumab plus chemotherapy in resectable ESCC, reporting a PCR rate of 41% with manageable safety profile, providing early evidence for the feasibility of neoadjuvant chemoimmunotherapy (NCIT) (6). More recently, the randomized phase 3 ESCORT-NEO/NCCES01 trial provided robust evidence by comparing neoadjuvant chemotherapy with or without camrelizumab in resectable ESCC. The results demonstrated that the addition of camrelizumab significantly improved PCR rates (28.0% vs 4.7%) without compromising surgical feasibility or increasing perioperative complications (7). Collectively, these studies indicate that neoadjuvant chemoimmunotherapy is rapidly reshaping the treatment paradigm for locally advanced ESCC, achieving unprecedented PCR rates and promising short-term outcomes.

The advent of the NCIT era, however, is accompanied by new challenges (8). Despite significantly improved PCR rates, the majority of patients still fail to achieve a PCR, and their prognoses exhibit substantial heterogeneity (9–11). Furthermore, for these patients who do not attain PCR after NCIT, whether adjuvant immunotherapy should be administered postoperatively to consolidate efficacy and, crucially, how to identify the subset that truly benefits from it, remain areas lacking high-level evidence, creating a critical blind spot in clinical decision-making (12, 13). Achieving precise prognostic stratification and tailored treatment is a central, unresolved issue in contemporary ESCC management.

Pathological evaluation serves as the cornerstone linking neoadjuvant treatment response to subsequent clinical decisions. Pathological complete response (PCR, ypT0N0) is universally recognized as a powerful predictor of superior long-term survival (14, 15). However, categorizing the NPCR majority as a single entity inevitably obscures the intrinsic heterogeneity within this group. Emerging evidence suggests that the anatomical distribution of residual disease (e.g., different combinations of primary tumor and nodal status) may harbor significant prognostic information (16–18). A key scientific question arises: can a refined subtyping system based on post-therapy pathology not only more accurately predict survival but also directly guide the precise application of adjuvant immunotherapy?

Therefore, this study aims to analyze a retrospective cohort of ESCC patients who underwent NCIT followed by surgery. We will first validate the prognostic value of PCR and then innovatively propose and validate a pathological subtyping system based on the anatomical distribution of residual disease. We will focus on investigating the differential responses to adjuvant immunotherapy across these subtypes. Ultimately, we will construct and validate prognostic nomograms for individualized survival prediction, seeking to provide a practical and actionable decision-making tool for the precision management of ESCC in the postoperative setting.

## 2 Patients and methods

### 2.1 Study population

#### 2.1.1 Ethical approval and patient enrollment

The study protocol was approved by the Institutional Review Board of Tianjin Medical University Cancer Institute and Hospital. Written informed consent was obtained from all participants. This study enrolled patients who received neoadjuvant chemoimmonotherapy followed by esophagectomy at our institution between June 2020 and June 2023. Prior to the study initiation date, neoadjuvant therapy was not routinely administered at our center. The surgical procedures and preoperative evaluation criteria have been previously described in the literature (19).

Locally advanced ESCC was defined as clinical stage cT2-4aN0M0 or cT1-4aN+M0 according to the AJCC 8th edition staging system. The inclusion criteria for this study were as follows: (1) patients with histologically confirmed esophageal squamous cell carcinoma (ESCC); (2) patients who received neoadjuvant chemoimmunotherapy; and (3) patients who underwent three-incision esophagectomy (McKeown procedure). The exclusion criteria included: (1) patients with non-squamous cell carcinoma pathology; (2) patients who underwent Ivor-Lewis esophagectomy due to inadequate lymph node dissection; (3) patients who received preoperative chemotherapy alone; and (4) patients who received radiotherapy.

### 2.2 Treatment protocols

In this study, all patients received neoadjuvant chemoimmunotherapy (NCIT) prior to surgery. The chemotherapy regimen consisted of paclitaxel plus platinum (TP regimen): Albumin-bound paclitaxel: (260 mg/m², intravenous infusion, over 30 minutes on Day 1 of each 21-day cycle) in combination with one of the following platinum-based agents: cisplatin (75 mg/m², intravenous infusion, over 2–3 hours on Day 1 of each 21-day cycle. Adequate hydration is required before and after cisplatin administration to prevent nephrotoxicity, typically consisting of at least 1–2 liters of normal saline or glucose-saline solution. Mannitol-induced diuresis may be used to enhance renal protection) or nedaplatin (80–100 mg/m², intravenous infusion, over 60 minutes on Day 1 of each 21-day cycle). Immunotherapy involved the use of a programmed cell death protein 1inhibitor (sintilimab/camrelizumab/pembrolizumab/tislelizumab, 200 mg/m², intravenous infusion, Day 1 of each 21-day cycle), administered concurrently with chemotherapy. Following surgical intervention, a subset of patients received adjuvant therapy in accordance with the study protocol. The adjuvant regimen consisted of monotherapy with a PD-1 inhibitor, without any concurrent chemotherapeutic agents.

### 2.3 Pathological evaluation

All surgical specimens were subjected to standardized pathological examination by two independent gastrointestinal pathologists who were blinded to the clinical data. Pathological complete response was strictly defined as the absence of any viable tumor cells in both the primary esophageal lesion and all resected regional lymph nodes (ypT0N0), in accordance with the American Joint Committee on Cancer (AJCC) 8th edition staging system. Major pathological response (MPR) was defined as ≤10% residual viable tumor in the primary tumor bed. The number of harvested lymph nodes and the presence of lymphovascular invasion were also documented for all specimens. Any discrepancies in pathological assessment were resolved through consensus review with a third senior pathologist.

### 2.4 Data collection and follow-up

Baseline demographic and clinical data were collected, including age, gender, tumor location, and clinical stage based on the 8th edition of the AJCC TNM staging system (20, 21). Additional prospectively collected data comprised details of neoadjuvant treatment regimens, the interval between completion of neoadjuvant therapy and surgery, and surgery-related parameters. Pathological evaluation included assessment of pathological complete response status, major pathological response (MPR) status, ypTNM stage, and the number of lymph nodes harvested. All postoperative complications were recorded and graded according to the Esophagectomy Complication Consensus Group (ECCG) criteria.

Patients were followed regularly postoperatively according to a standardized protocol: every 3 months for the first 2 years, every 6 months for years 3 to 5, and annually thereafter. Follow-up assessments included detailed medical history, physical examination, laboratory tests, cervical ultrasound, and contrast-enhanced computed tomography (CT) scans of the chest and abdomen. When clinically indicated, upper gastrointestinal contrast studies, positron emission tomography-computed tomography (PET-CT), and endoscopy were performed. DFS was calculated from the date of surgery to the date of disease recurrence or death from any cause. OS was defined as the time from surgery to death from any cause. Locoregional recurrence was defined as recurrence at the anastomotic site or within the regional lymph node basin, while distant metastasis referred to metastasis to organs or lymph nodes beyond these anatomical boundaries.

### 2.5 Statistical analysis

Categorical variables were presented as frequencies and percentages and compared using the Chi-square test or Fisher’s exact test, as appropriate. Survival curves for DFS and OS were generated using the Kaplan-Meier method and compared with the log-rank test. A Cox proportional hazards regression model was used for univariate and multivariate analyses to identify independent prognostic factors. Variables with a p-value < 0.1 in the univariate analysis were included in the multivariate model. A two-sided p-value < 0.05 was considered statistically significant. Statistical analyses and visualizations for the prognostic nomogram were conducted in R software (version 4.2.1) utilizing the survival package (version 3.4-0) for Cox regression analysis with proportional hazards assumption testing and the rms package (version 6.3-0) for nomogram construction and graphical presentation. All descriptive statistics, univariate and multivariate Cox regression analyses were performed using SPSS version 25.0. Nomogram construction, calibration plots, and C-index calculations were conducted using R software (version 4.2.1) with the rms and survival packages.

## 3 Results

### 3.1 Patient characteristics and pathological response

A total of 243 patients with locally advanced ESCC who received neoadjuvant therapy were included in this study. Among them, 67 patients (27.6%) achieved a pathological complete response (PCR group), while 176 patients (72.4%) did not (NPCR group). A comparison of the baseline clinical characteristics between the two groups is detailed in **Table 1**.

**Table 1:** Baseline clinicopathological characteristics of the study cohor.

Characteristics	Total (n=243)	PCR (n=67)	NPCR (n=176)	P value	T+N+ (n=88)	P value	T+N0 (n=56)	P value	T0N+ (n=32)	P value
**Age (%)**				0.155		0.418		0.949		0.494
<60	88 (36.2)	19 ( 28.4)	107 (60.8)		37 (42.0)		18 (32.1)		14 (43.8)	
≥60	155 (63.8)	48 ( 71.6)	69 (39.2)		51 (58.0)		38 (67.9)		18 (56.2)	
**Sex (%)**			0.314			0.031		1		1
Male	210 (86.4)	55 ( 82.1)	155 (88.1)		5 (5.7)		10 (17.9)		26 (81.2)	
Female	33 (13.6)	12 ( 17.9)	21 (11.9)		83 (94.3)		46 (82.1)		6 (18.8)	
**Tumor location (%)**				0.348		0.248		0.354		0.312
Upper	28 (11.5)	10 ( 14.9)	18 (10.2)		10 (11.4)		4 (7.1)		4 (12.5)	
Middle	93 (38.3)	28 ( 41.8)	65 (36.9)		28 (31.8)		28 (50.0)		9 (28.1)	
Lower	122 (50.2)	29 ( 43.3)	93 (52.8)		50 (56.8)		24 (42.9)		19 (59.4)	
**Drinking (%)**				0.775		0.299		1		0.586
Never	89 (36.6)	26 ( 38.8)	63 (35.8)		26 (29.5)		22 (39.3)		15 (46.9)	
Ever	154 (63.4)	41 ( 61.2)	113 (64.2)		62 (70.5)		34 (60.7)		17 (53.1)	
**Smoking (%)**				0.665		0.783		0.382		0.904
Never	98 (40.3)	29 ( 43.3)	69 (39.2)		35 (39.8)		19 (33.9)		15 (46.9)	
Ever	145 (59.7)	38 ( 56.7)	107 (60.8)		53 (60.2)		37 (66.1)		17 (53.1)	
**cT stage (%)**				0.353		0.227		0.993		0.146
T1	5 (2.0)	3 ( 4.5)	2 ( 1.1)		0 (0.0)		2 (3.6)		0 (0.0)	
T2	7 (2.9)	1 ( 1.5)	6 ( 3.4)		2 (2.3)		1 (1.8)		3 (9.4)	
T3	198 (81.5)	54 ( 80.6)	144 (81.8)		76 (86.4)		45 (80.4)		23 (71.9)	
T4	33 (13.6)	9 ( 13.4)	24 (13.6)		10 (11.4)		8 (14.3)		6 (18.8)	
**cN stage (%)**				0.208		0.011		0.982		0.164
N0	34 (14.0)	14 ( 20.9)	20 (11.4)		4 (4.5)		12 (21.4)		4 (12.5)	
N1	124 (51.0)	32 ( 47.8)	92 (52.3)		53 (60.2)		28 (50.0)		11 (34.4)	
N2	67 (27.6)	15 ( 22.4)	52 (29.5)		26 (29.5)		12 (21.4)		14 (43.8)	
N3	18 (7.4)	6 ( 9.0)	12 ( 6.8)		5 (5.7)		4 (7.1)		3 (9.4)	
**cTNM stage (%)**				0.44		0.037		0.986		0.988
II	24 (9.9)	9 ( 13.4)	15 ( 8.5)		3 (3.4)		8 (14.3)		4 (12.5)	
III	172 (70.8)	44 ( 65.7)	128 (72.7)		71 (80.7)		36 (64.3)		21 (65.6)	
IV	47 (19.3)	14 ( 20.9)	33 (18.8)		14 (15.9)		12 (21.4)		7 (21.9)	
**ypT stage (%)**				<0.001		<0.001		<0.001		NA
T0	100 (41.2)	67 (100.0)	33 (18.8)		0 (0.0)		0 (0.0)		32 (100.0)	
T1	46 (18.9)	0 ( 0.0)	46 (26.1)		18 (20.5)		29 (51.8)		0 (0.0)	
T2	39 (16.1)	0 ( 0.0)	39 (22.2)		27 (30.7)		12 (21.4)		0 (0.0)	
T3	55 (22.6)	0 ( 0.0)	55 (31.2)		42 (47.7)		13 (23.2)		0 (0.0)	
T4	3 (1.2)	0 ( 0.0)	3 ( 1.7)		1 (1.1)		2 (3.6)		0 (0.0)	
**ypN stage (%)**				<0.001		<0.001		NA		<0.001
N0	123 (50.6)	67 (100.0)	56 (31.8)		0 (0.0)		56 (100.0)		0 (0.0)	
N1	64 (26.3)	0 ( 0.0)	64 (36.4)		39 (44.3)		0 (0.0)		25 (78.1)	
N2	41 (16.9)	0 ( 0.0)	41 (23.3)		37 (42.0)		0 (0.0)		4 (12.5)	
N3	15 (6.2)	0 ( 0.0)	15 ( 8.5)		12 (13.6)		0 (0.0)		3 (9.4)	
**ypTNM stage (%)**				<0.001		<0.001		<0.001		NA
/		67 (100.0)	32 (19.2)		0 (0.0)		0 (0.0)		32 (100.0)	
I		0 ( 0.0)	29 (17.4)		0 (0.0)		29 (51.8)		0 ( 0.0)	
II		0 ( 0.0)	36 (21.6)		11 (12.5)		25 (44.6)		0 ( 0.0)	
III		0 ( 0.0)	66 (39.5)		64 (72.7)		2 (3.6)		0 ( 0.0)	
IV		0 ( 0.0)	13 (7.8)		13 (14.8)		0 (0.0)		0 ( 0.0)	

pCR, pathological complete response; NPCR, non-pathological complete response; cT, clinical T stage; cN, clinical N stage; cTNM, clinical TNM stage; ypT, post-neoadjuvant pathological T stage; ypN, post-neoadjuvant pathological N stage; ypTNM, post-neoadjuvant pathological TNM stage. P-values refer to comparisons between each subgroup and the pCR group.

The median age of the overall cohort was 63 years (range, 39–77 years). Regarding demographic features, the proportion of patients aged ≥60 years was comparable between the PCR group and the NPCR group (71.6% vs 39.2%, P > 0.05), with no significant imbalance observed for this variable. No statistically significant differences were observed between the two groups in terms of sex, drinking history, or smoking history (P > 0.05 for all).

Analysis of tumor-related characteristics revealed no significant differences in the distribution of tumor location, clinical T stage (cT), clinical N stage (cN), or clinical TNM stage between the two groups (P > 0.05 for all), suggesting a generally balanced baseline tumor burden.

### 3.2 Survival outcomes

**Figure 1:**
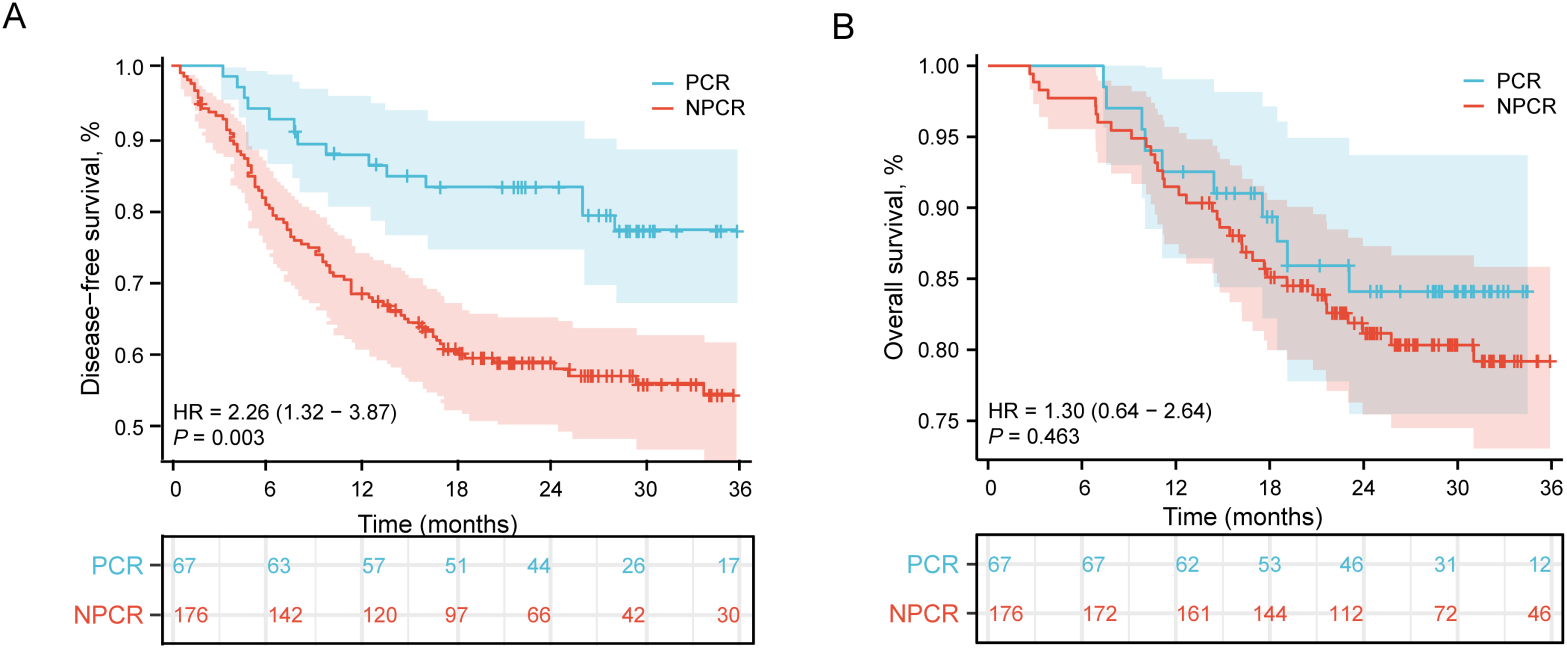
Comparison of survival outcomes between patients achieving pCR and those with NPCR. **(A)** Kaplan-Meier curves for DFS. Patients in the pCR group showed significantly improved DFS compared to the NPCR group (HR = 2.26, 95% CI: 1.32-3.87, P = 0.003). **(B)** Kaplan-Meier curves for OS. Although the pCR group demonstrated better OS, the difference was not statistically significant (HR = 1.30, 95% CI: 0.64-2.64, P = 0.463). Numbers at risk at each time point are shown below the corresponding panels.

Patients achieving pathological complete response demonstrated distinct survival outcomes compared to NPCR patients following neoadjuvant chemoimmunotherapy and esophagectomy. As presented in **Figure 1A**, the PCR group showed significantly improved DFS compared to the NPCR group (hazard ratio [HR] = 2.26, 95% confidence interval [CI]: 1.32-3.87, P = 0.003), indicating that PCR status was associated with a substantially reduced risk of disease recurrence or death. Regarding overall survival outcomes (**Figure 1B**), although the PCR group exhibited better survival trends, the difference did not reach statistical significance (HR = 1.30, 95% CI: 0.64-2.64, P = 0.463). In patients achieving pCR, the median DFS was 27.8 months in the adjuvant therapy group versus 28.8 months in the observation group, and the median OS was 31.7 months versus 28.8 months, with no statistically significant differences between the two groups for either DFS or OS.

### 3.3 Stratified survival analysis based on treatment modalities in PCR patients

**Figure 2:**
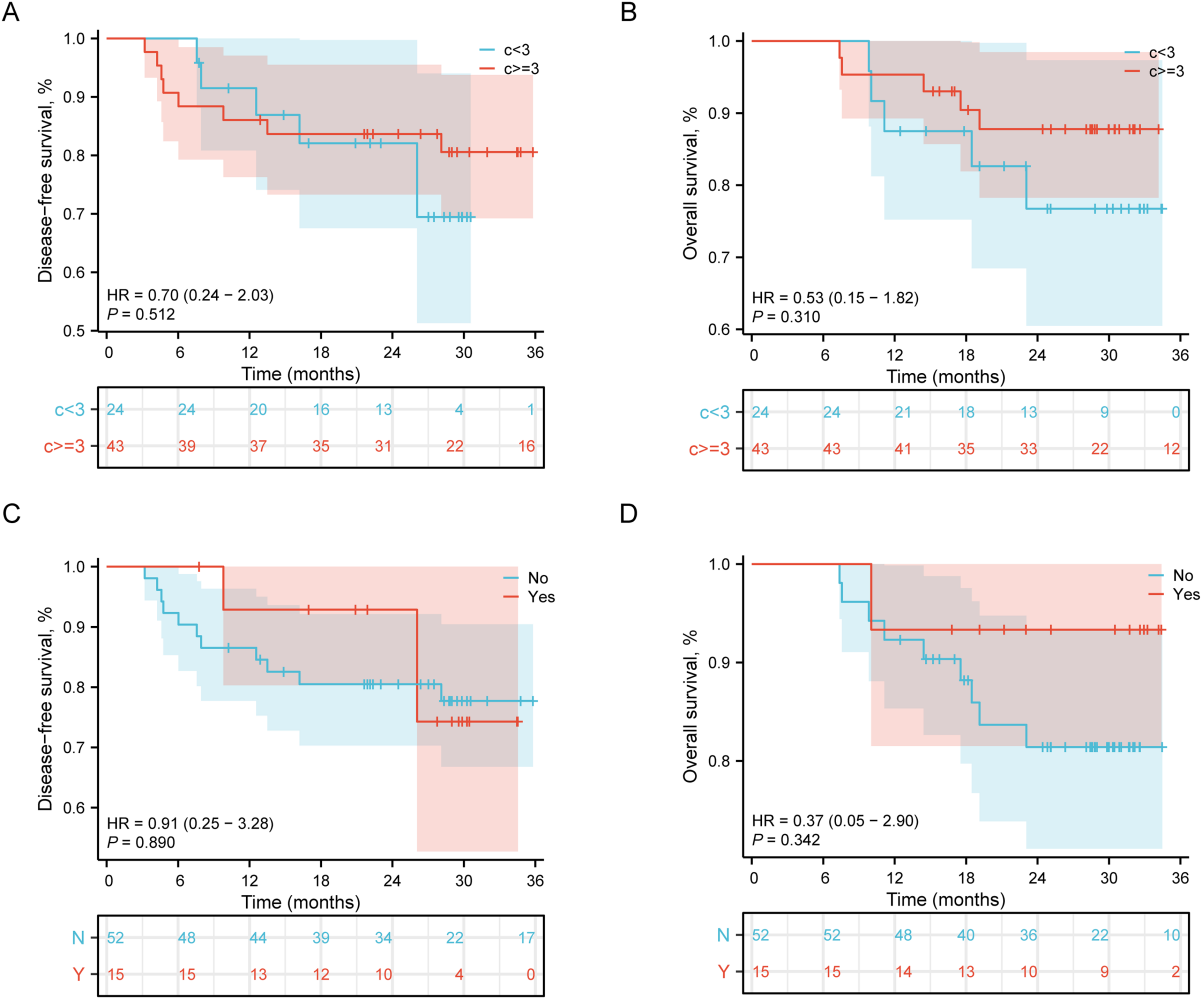
Survival outcomes stratified by treatment modalities in patients achieving pCR. **(A)** DFS by number of neoadjuvant therapy cycles (<3 vs ≥3 cycles). Hazard ratio (HR) = 0.70, 95% confidence interval (CI): 0.24-2.03, P = 0.512. **(B)** OS by number of neoadjuvant therapy cycles (<3 vs ≥3 cycles). HR = 0.53, 95% CI: 0.15-1.82, P = 0.310. **(C)** DFS by adjuvant therapy status (Yes vs No). HR = 0.91, 95% CI: 0.25-3.28, P = 0.890. **(D)** OS by adjuvant therapy status (Yes vs No). HR and P values are consistent with DFS analysis in panel **C**. Numbers at risk at each time point are shown below each corresponding panel.

Among patients who achieved pathological complete response, subsequent treatment parameters demonstrated no significant impact on survival outcomes. As shown in **Figures 2A, B**, stratification by the number of neoadjuvant therapy cycles revealed comparable disease-free survival (DFS: HR = 0.70, 95% CI: 0.24-2.03, P = 0.512) and overall survival (OS: HR = 0.53, 95% CI: 0.15-1.82, P = 0.310) between patients receiving <3 versus ≥3 cycles. Similarly, **Figures 2C, D** illustrates that the administration of adjuvant immunotherapy in PCR patients failed to demonstrate significant survival benefits, with comparable DFS (HR = 0.91, 95% CI: 0.25-3.28, P = 0.890) and OS outcomes between treatment groups.

### 3.4 Stratified survival analysis in NPCR patients

**Figure 3:**
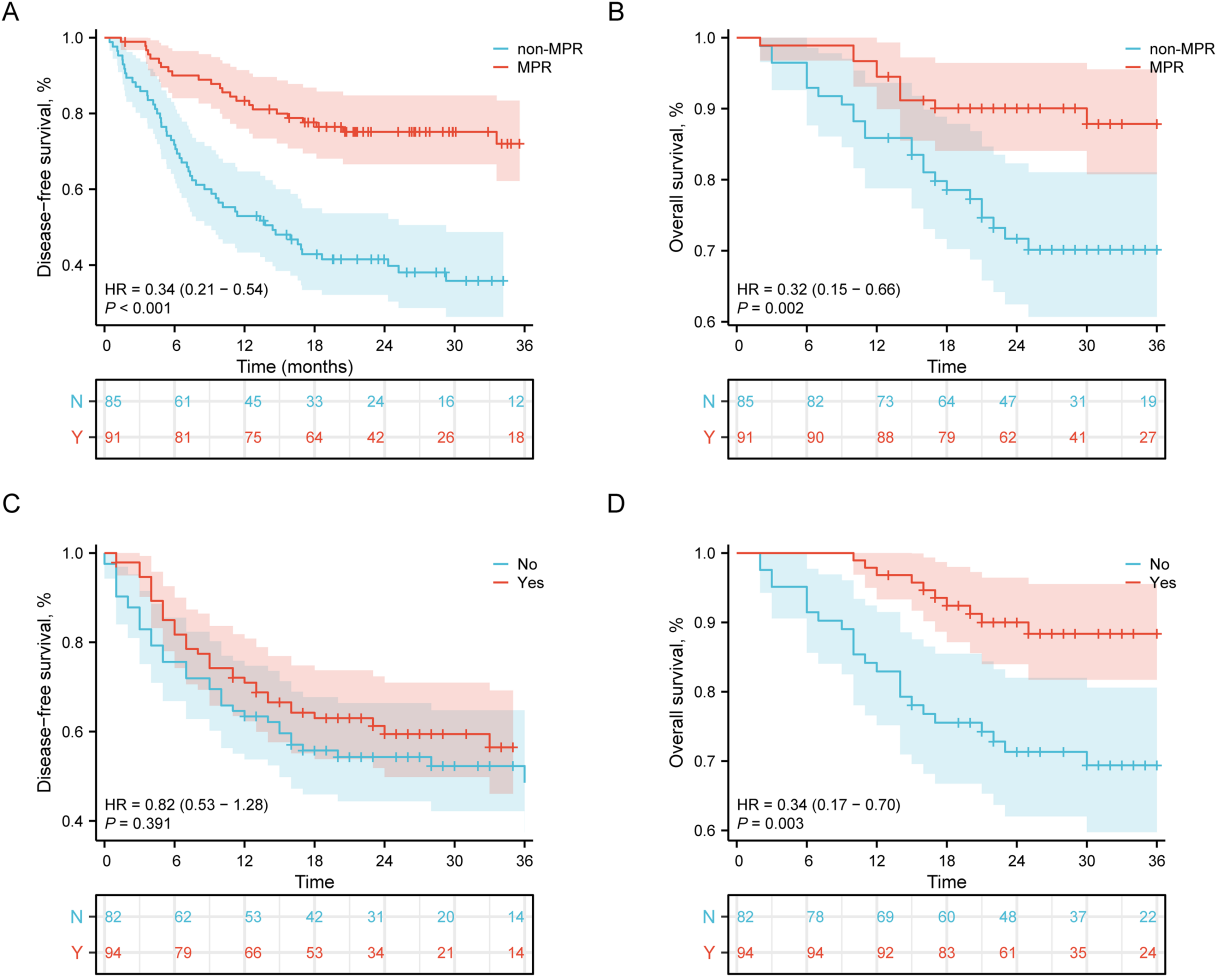
Survival outcomes in NPCR patients stratified by pathological response and adjuvant therapy. **(A)** DFS by MPR status in NPCR patients. Patients achieving MPR showed significantly improved DFS compared to those without MPR (HR = 0.34, 95% CI: 0.21-0.54, P < 0.001). **(B)** OS by MPR status in NPCR patients. The MPR group demonstrated significantly better OS outcomes (HR = 0.32, 95% CI: 0.15-0.66, P = 0.002). **(C)** DFS by adjuvant therapy in NPCR patients. No significant DFS benefit was observed with adjuvant therapy (HR = 0.82, 95% CI: 0.53-1.28, P = 0.391). **(D)** OS by adjuvant therapy in NPCR patients. Adjuvant therapy was associated with significantly improved OS (HR = 0.34, 95% CI: 0.17-0.70, P = 0.003). Numbers at risk at each time point are shown below the corresponding panels.

Survival analysis based on pathological response and adjuvant therapy status in NPCR esophageal squamous cell carcinoma patients revealed clinically significant findings. As shown in **Figure 3A**, the disease-free survival assessment using Kaplan-Meier curves demonstrated that patients achieving major pathological response maintained significantly superior disease control during the 36-month follow-up period, with a 66% reduction in risk of disease recurrence or death compared to non-MPR patients. The overall survival analysis in **Figure 3B** further confirmed this trend, showing sustained survival advantage for MPR patients throughout the observation period with a statistically significant hazard ratio of 0.32 (HR = 0.32, 95% CI: 0.15-0.66, P = 0.002).

In evaluating the efficacy of postoperative adjuvant therapy, **Figure 3C** shows that among NPCR patients, no statistically significant difference in DFS was observed between those who received adjuvant therapy and those who did not (HR = 0.82, 95% CI: 0.53-1.28, P = 0.391). In contrast, the OS analysis in **Figure 3D** demonstrated a statistically significant advantage for the adjuvant therapy group, with a 66% reduction in the risk of death (HR = 0.34, 95% CI: 0.17-0.70, P = 0.003).

### 3.5 Efficacy of adjuvant immunotherapy stratified by pathological subtypes in NPCR patients

Building upon the established prognostic value of major pathological response (MPR) status in the overall NPCR cohort (**Figures 3A, B**) and the differential effects of adjuvant therapy on OS versus DFS (**Figures 3C, D**), we conducted further stratified analyses to evaluate potential heterogeneity in treatment response. NPCR patients were systematically categorized into three distinct pathological subgroups based on the anatomical distribution of residual disease following neoadjuvant therapy: the T+N+ subgroup (residual tumor present in both primary site and regional lymph nodes), the T+N0 subgroup (residual tumor strictly confined to the primary site), and the T0N+ subgroup (residual disease limited to regional lymph nodes only).

**Figure 4:**
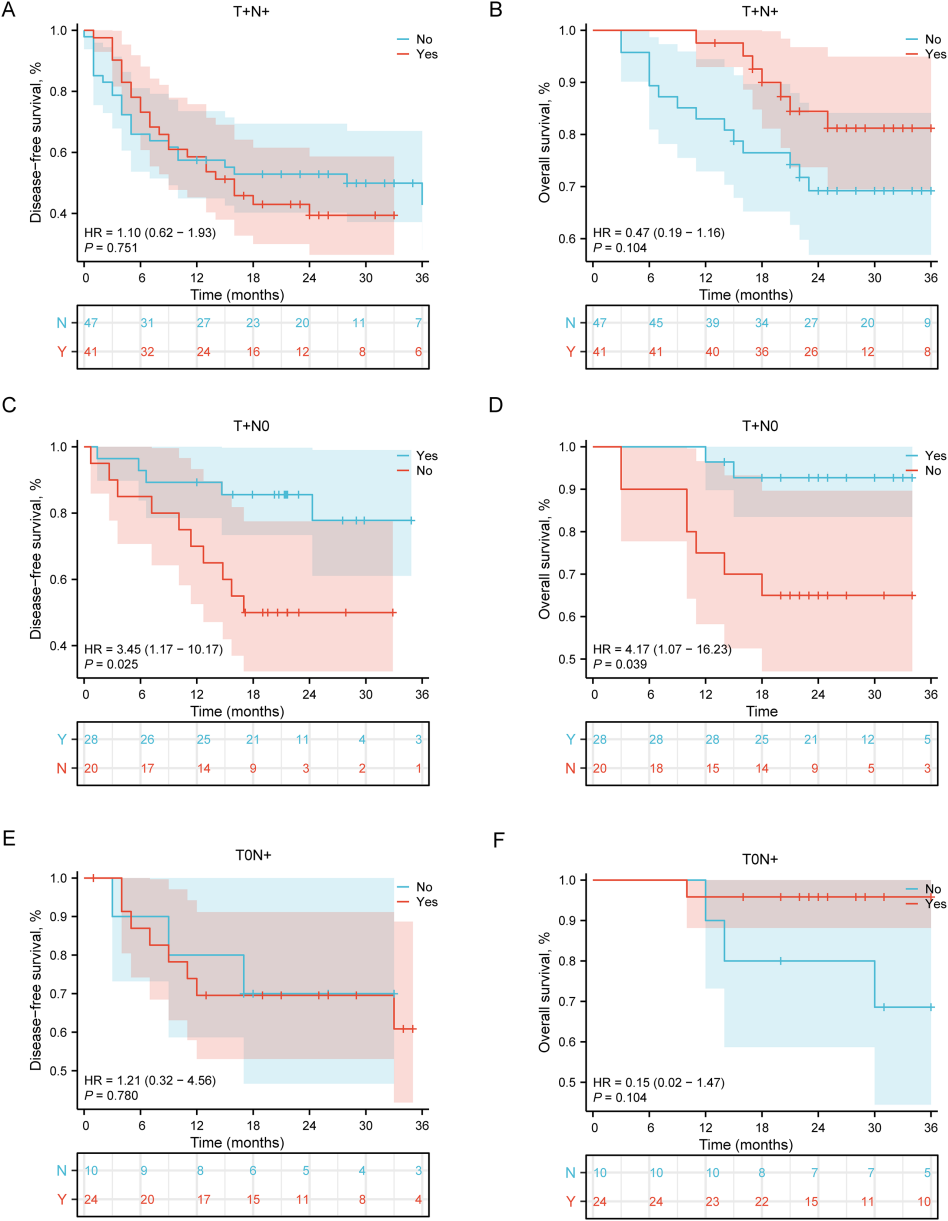
Impact of adjuvant therapy on survival outcomes in NPCR patients stratified by pathological subtypes. **(A)** DFS in T+N+ subgroup (residual tumor in both primary site and lymph nodes). HR = 1.10, 95% CI: 0.62-1.93, P = 0.751. **(B)** Overall survival in T+N+ subgroup. HR = 0.47, 95% CI: 0.19-1.16, P = 0.104. **(C)** Disease-free survival in T+N0 subgroup (residual tumor in primary site only). HR = 3.45, 95% CI: 1.17-10.17, P = 0.025. **(D)** Overall survival in T+N0 subgroup. HR = 4.17, 95% CI: 1.07-16.23, P = 0.039. **(E)** Disease-free survival in T0N+ subgroup (residual disease in lymph nodes only). HR = 1.21, 95% CI: 0.32-4.56, P = 0.780. **(F)** Overall survival in T0N+ subgroup. HR = 0.15, 95% CI: 0.02-1.47, P = 0.104. Numbers at risk at each time point are shown below the corresponding panels. T+N+: residual tumor in both primary site and lymph nodes; T+N0: residual tumor in primary site only; T0N+: residual disease in lymph nodes only.

In the T+N+ subgroup (**Figures 4A, B**), representing patients with the highest residual tumor burden after neoadjuvant therapy, adjuvant immunotherapy failed to demonstrate statistically significant survival benefits. DFS analysis revealed a hazard ratio (HR) of 1.10 (95% confidence interval [CI]: 0.62-1.93, P = 0.751), indicating no significant difference in recurrence risk between treatment and control groups. Concurrently, OS analysis yielded an HR of 0.47 (95% CI: 0.19-1.16, P = 0.104), while numerically favoring the treatment group, did not reach statistical significance.

Notably, in the T+N0 subgroup (residual tumor strictly confined to the primary site; **Figures 4C, D**), adjuvant immunotherapy demonstrated substantial clinical benefits. DFS analysis produced an HR of 3.45 (95% CI: 1.17-10.17, P = 0.025), indicating a significantly reduced risk of recurrence among patients receiving adjuvant immunotherapy. Correspondingly, OS analysis showed an HR of 4.17 (95% CI: 1.07-16.23, P = 0.039), demonstrating significantly prolonged overall survival in this subgroup.

In the T0N+ subgroup (residual disease limited to regional lymph nodes; **Figures 4E, F**), adjuvant immunotherapy demonstrated no significant survival benefit, with a DFS HR of 1.21 (95% CI: 0.32–4.56, P = 0.780) and an OS HR of 0.15 (95% CI: 0.02–1.47, P = 0.104).

### 3.6 Comparative survival analysis between pathological subtypes of NPCR patients and the PCR cohort

To precisely delineate the prognostic implications of different residual disease patterns, we performed a direct survival comparison between each NPCR pathological subtype and the PCR cohort, which served as the reference benchmark.

**Figure 5:**
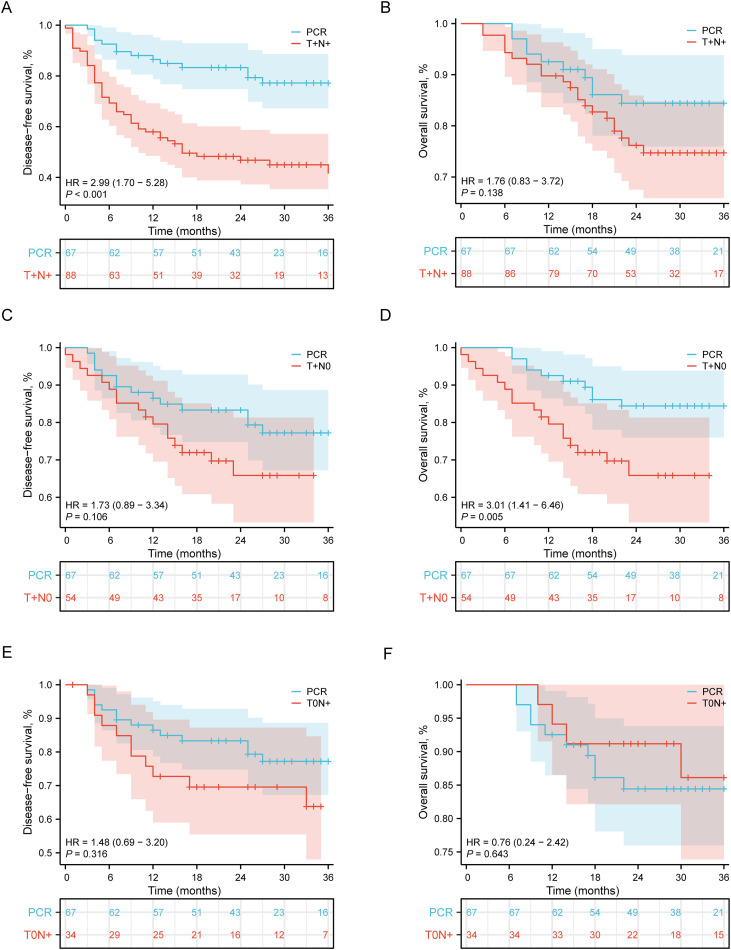
Comparison of Survival Outcomes between pCR and NPCR Patients Stratified by Residual Disease Patterns. **(A)** DFS in pCR vs. T+N+ patients. Patients with residual tumor in both the primary site and lymph nodes (T+N+) showed significantly inferior DFS compared to those achieving pCR (Hazard Ratio [HR] = 2.99, 95% Confidence Interval [CI]: 1.70–5.28; P < 0.001). **(B)** OS in pCR vs. T+N+ patients. No statistically significant difference in OS was observed between the T+N+ subgroup and the pCR group (HR = 1.76, 95% CI: 0.83–3.72; P = 0.138). **(C)** DFS in pCR vs. T+N0 patients. The DFS of patients with residual tumor confined to the primary site only (T+N0) was not significantly different from that of the pCR group (HR = 1.71, 95% CI: 0.89–3.34; P = 0.106). **(D)**. OS in pCR vs. T+N0 patients. Patients in the T+N0 subgroup had significantly worse OS compared to patients who achieved pCR (HR = 3.01, 95% CI: 1.41–6.46; P = 0.005). **(E)** DFS in pCR vs. T0N+ patients. DFS was comparable between patients with residual disease limited to lymph nodes only (T0N+) and the pCR group (HR = 1.48, 95% CI: 0.69–3.20; P = 0.316). **(F)** OS in pCR vs. T0N+ patients. The OS of the T0N+ subgroup was not significantly different from that of the pCR group (HR = 0.76, 95% CI: 0.24–2.42; P = 0.643). The number of patients at risk at each time point is shown below the corresponding panels.

Among the three non-pCR subtypes, the T+N+ subgroup had the worst prognosis, with significantly inferior DFS compared to the pCR group. Their DFS was significantly inferior (HR = 2.99, 95% CI: 1.70–5.28; P < 0.001; **Figure 5A**). A consistent trend towards worse OS was observed, although the difference was not statistically significant (HR = 1.76, 95% CI: 0.83–3.72; P = 0.138; **Figure 5B**).

A distinct, dissociated survival pattern was observed in the T+N0 subgroup (residual tumor confined to the primary site). While their DFS was not significantly different from that of PCR patients (HR = 1.71, 95% CI: 0.89–3.34; P = 0.106; **Figure 5C**), they demonstrated significantly poorer OS (HR = 3.01, 95% CI: 1.41–6.46; P = 0.005; **Figure 5D**).

Conversely, the survival outcomes of the T0N+ subgroup (residual disease limited to lymph nodes) most closely approximated those of the PCR benchmark. No significant differences were found between the T0N+ and PCR groups for either DFS (HR = 1.48, 95% CI: 0.69–3.20; P = 0.316; **Figure 5E**) or OS (HR = 0.76, 95% CI: 0.24–2.42; P = 0.643; **Figure 5F**).

### 3.7 Construction and validation of prognostic nomograms

Building upon the identification of key prognostic factors—including pathological response status, post-therapy pathological stage, and clinical staging—we sought to integrate these variables into a practical tool for individualized risk assessment. Consequently, we developed two prognostic nomograms based on the results of the multivariate Cox regression analysis.

**Figure 6:**
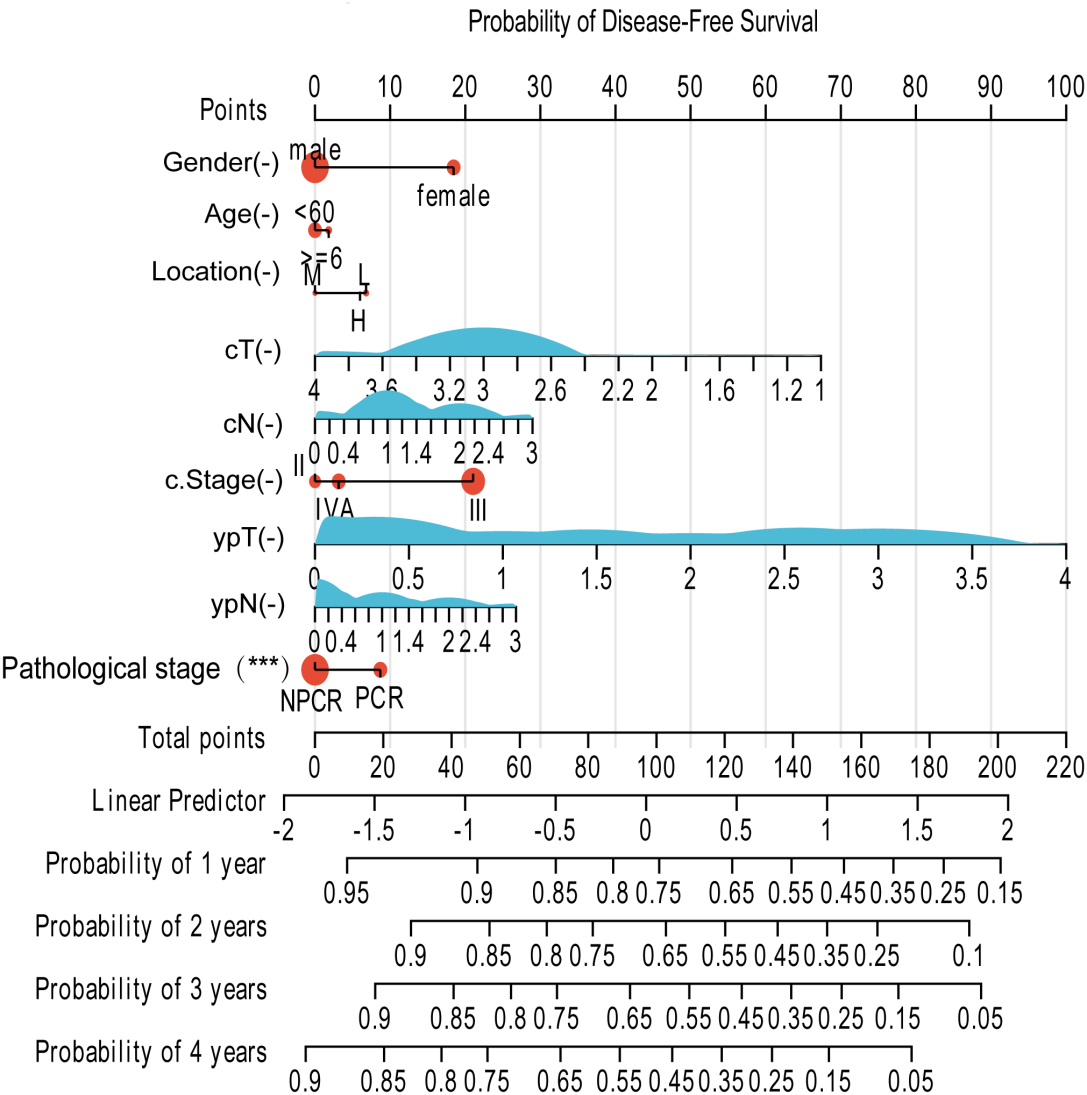
Prognostic nomogram for predicting DFS in patients with esophageal squamous cell carcinoma following neoadjuvant therapy. The nomogram predicts the probability of DFS at 1, 2, 3, and 4 years. This model was constructed using the following clinicopathological variables: Gender, Age, Tumor Location, clinical T stage (cT), clinical N stage (cN), clinical overall stage (c.Stage), post-neoadjuvant pathological T stage (ypT), post-neoadjuvant pathological N stage (ypN), and Pathological Response (PCR vs. NPCR). To utilize the nomogram, for each variable, locate the patient's value on the corresponding axis and draw a vertical line upward to the 'Points' axis to determine the score. Sum the points for all variables to obtain the 'Total Points'. Finally, draw a vertical line downward from the 'Total Points' axis to the survival probability axes at the bottom to estimate the likelihood of DFS at each time point.

The first nomogram (**Figure 6**) integrates significant clinicopathological variables to predict the probability of DFS at 1, 2, 3, and 4 years after treatment. The model incorporates key predictors including patient gender, age, tumor location, clinical T stage (cT), clinical N stage (cN), clinical stage (c.Stage), post-therapy pathological T stage (ypT), post-therapy pathological N stage (ypN), and most notably, the pathological state (PCR vs. NPCR). Each patient’s profile can be converted into a total point score, which corresponds to a specific probability of DFS.

**Figure 7:**
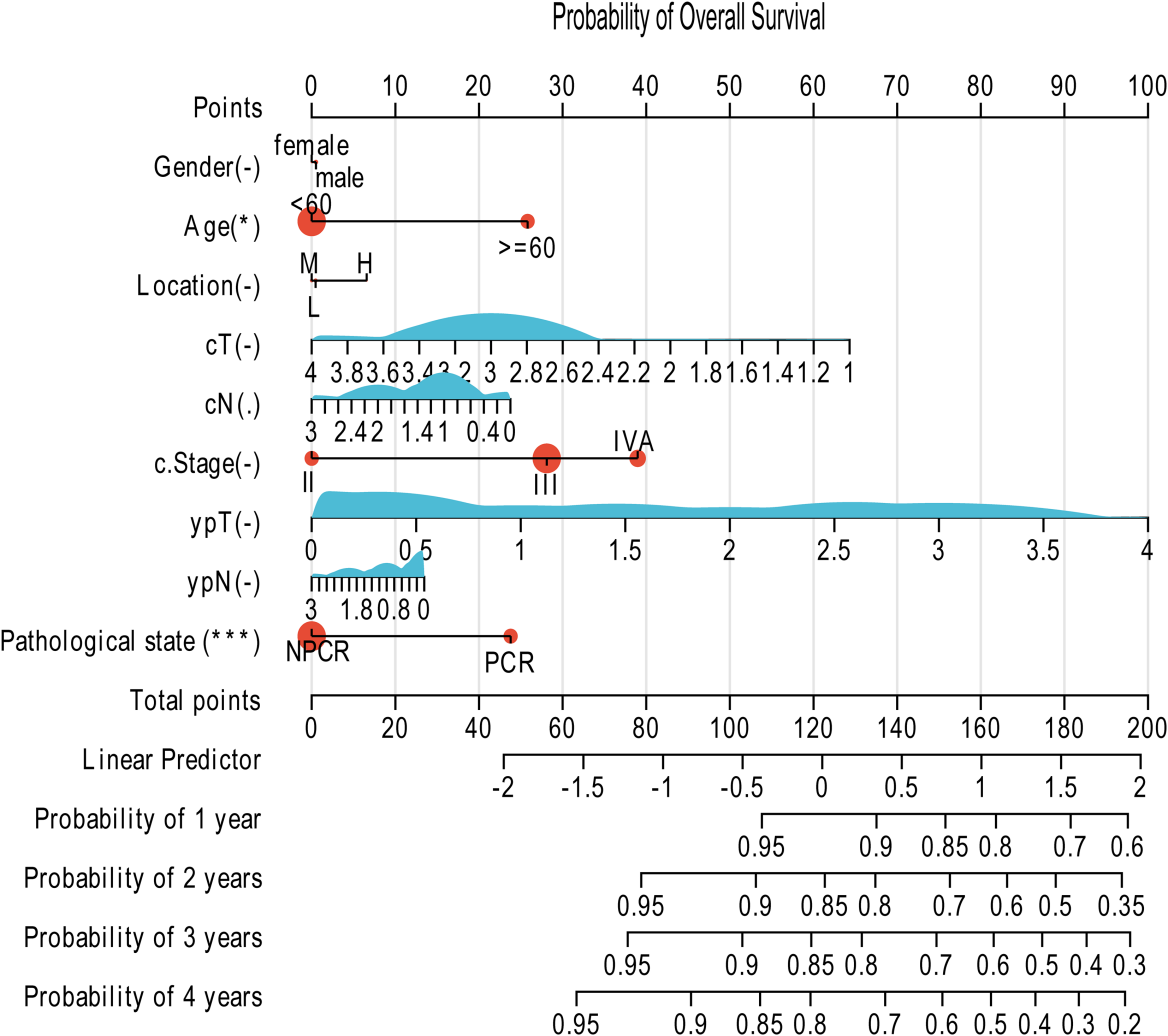
Prognostic nomogram for predicting OS in patients with esophageal squamous cell carcinoma following neoadjuvant therapy. The nomogram predicts the probability of OS at 1, 2, 3, and 4 years. This model incorporates the same set of predictors as the DFS nomogram: Gender, Age, Tumor Location, cT stage, cN stage, c.Stage, ypT stage, ypN stage, and Pathological state (PCR vs. NPCR). The application method is identical to that of **Figure 6**: assign points for each variable based on the patient's data, sum these points to calculate the total score, and then project the total score downward to the survival axes to determine the predicted probabilities of OS.

Similarly, the second nomogram (**Figure 7**) was constructed to predict the probability of OS at the same timepoints. It utilizes the same set of robust predictors, allowing for a direct, visual estimation of individual survival likelihood based on the cumulative points derived from all included variables.

Both nomograms demonstrated good predictive accuracy upon internal validation, with a bootstrap-corrected concordance index (C-index) exceeding 0.75 for both DFS and OS predictions, indicating a reliable performance in stratifying patients according to their recurrence and mortality risks. In summary, these validated nomograms provide a user-friendly and quantitative means for clinicians to estimate postoperative survival probabilities, thereby potentially informing personalized follow-up strategies and adjuvant therapy decisions.

## 4 Discussion

The treatment landscape for locally advanced esophageal squamous cell carcinoma (ESCC) has undergone significant transformation over the past decade. While neoadjuvant chemoradiotherapy (NCRT) followed by esophagectomy, established by the CROSS trial paradigm, represented the previous standard of care (22), the advent of immune checkpoint inhibitors has fundamentally reshaped therapeutic strategies. The combination of PD-1 inhibitors with chemotherapy in the neoadjuvant setting—neoadjuvant chemoimmunotherapy (NCIT)—has demonstrated remarkable efficacy, achieving pathological complete response rates substantially higher than those observed with chemotherapy or chemoradiotherapy alone (23, 24). This paradigm shift towards NCIT constitutes a major advancement in ESCC management. However, the consequent clinical challenge has shifted to optimizing postoperative strategies for the majority of patients who do not achieve PCR, creating an urgent need for biomarkers to guide adjuvant therapy decisions.

The role of adjuvant therapy in this new context remains particularly contentious. Although adjuvant immunotherapy has demonstrated survival benefits in several solid malignancies, its utility in ESCC patients who have already received NCIT is not well-defined. The pivotal clinical question has evolved beyond simply whether to administer adjuvant therapy, to how to identify the specific patient subsets that would derive genuine benefit from it, thereby avoiding unnecessary treatment-related toxicity and healthcare costs for those who would not.

Within this context, our study provides crucial evidence to address this knowledge gap. Our data robustly reaffirm PCR as a powerful surrogate for superior survival, with the near-doubling of 3-year DFS in PCR patients aligning with previous studies evaluating novel neoadjuvant regimens (25). However, the critical advancement of our work lies in moving beyond the binary PCR/NPCR paradigm. By introducing a simple yet highly discriminative classification system based on the anatomical distribution of residual disease (T+N+, T+N0, and T0N+) we have successfully deconvoluted the heterogeneity within the NPCR population.

The marked prognostic gradient observed among these subtypes is striking. The T+N+ subtype, characterized by residual disease in both anatomical compartments, exhibited the poorest prognosis, likely reflecting an aggressive tumor biology with systemic dissemination potential and a profoundly immunosuppressive tumor microenvironment (TME) that persists after NCIT (26, 27). It is noteworthy that although T0N+ patients showed no statistical difference from the pCR group, the sample size is small, and the curves show a trend of separation, so conclusions should not be drawn hastily. For T0N+ patients, surgery alone may have achieved effective disease control; however, whether adjuvant therapy can be safely omitted in this subgroup requires further validation with more clinical data.

The most compelling and clinically translatable insight from our analysis pertains to the differential efficacy of adjuvant immunotherapy across these pathological subtypes. The current clinical dilemma often involves a “one-size-fits-all” consideration of adjuvant therapy for NPCR patients. Our findings fundamentally challenge this approach. We identified the T+N0 subtype—patients with residual tumor confined to the primary site but cleared from lymph nodes—as the primary beneficiary of adjuvant immunotherapy, showing significant improvements in both DFS and OS. This suggests a unique biological context: the primary TME in these patients may retain sufficient immunologic responsiveness or harbor a distinct immune cell repertoire that can be effectively reinvigorated by PD-1 inhibition to eradicate minimal residual disease and micrometastatic seeds originating from the primary site (28, 29).

Conversely, the lack of significant benefit from adjuvant immunotherapy in the T+N+ subgroup is equally informative. The extensive residual burden in both primary and nodal sites may signify a deeply entrenched, therapy-resistant biology (30). The TME in these patients is likely dominated by exclusionary or suppressive mechanisms that render the tumor insensitive to further immune checkpoint blockade. For this high-risk group, our data suggest that merely adding adjuvant immunotherapy is insufficient. Alternative strategies, such as novel drug combinations based on molecular profiling, targeted therapies, or early enrollment in clinical trials investigating innovative approaches, should be prioritized.

No significant survival benefit was observed in the T0N+ subgroup. Although these patients demonstrated a relatively favorable prognosis, with survival curves approximating those of the pCR population, this finding should be interpreted with caution given the limited sample size. Whether the absolute benefit of adding adjuvant immunotherapy is truly limited for T0N+ patients remains uncertain. Therefore, the adoption of a close surveillance strategy to omit adjuvant therapy in this subtype requires further validation through larger prospective studies. The current data provide only preliminary insights for clinical decision-making and are insufficient to support changes to standard treatment practice.

To facilitate the translation of these findings into clinical practice, we developed and internally validated prognostic nomograms that integrate the pathological subtype with other key clinicopathological variables. These user-friendly tools provide a quantitative means for individualized risk estimation, aiding shared decision-making regarding postoperative management.

Our study has several limitations. First, its retrospective, single-center design introduces potential selection bias. Second, the relatively small sample sizes of certain subtypes, particularly T0N+, limit the statistical power of some comparisons; therefore, these findings require validation in larger prospective cohorts. Although the median follow-up of 36 months provides valuable information, it remains relatively short for definitive conclusions regarding overall survival in esophageal squamous cell carcinoma. It is important to emphasize that the lack of significant benefit observed in the T+N+ and T0N+ subtypes does not imply that immunotherapy is ineffective in these populations, but rather suggests that monotherapy may be insufficient to overcome residual tumor burden under the current regimen. Future studies should evaluate more intensive or rationally combined adjuvant strategies (e.g., immunotherapy plus chemotherapy) in these high-risk groups. In addition, we acknowledge that the absence of adjuvant chemotherapy in our study design may have underestimated the potential benefit in certain subgroups, a limitation that has been explicitly addressed in the Discussion. Finally, the molecular underpinnings driving the distinct behaviors and differential treatment responses across these pathological subtypes remain unexplored, representing a critical direction for future research. Analyzing the genomic, transcriptomic, and immune contexture of residual tumors in these subtypes may uncover novel biomarkers and therapeutic targets. In summary, we interpret the findings related to the T0N+ subtype with caution, emphasizing the need for careful validation in larger cohorts. For all node-positive patients, single-agent adjuvant therapy may be insufficient, and future trials should investigate more intensive regimens in this population.

In conclusion, we propose a novel, practical, and robust pathological subtyping framework for managing NPCR ESCC patients after NCIT. This framework not only provides superior prognostic stratification but also, for the first time, identifies a specific patient population (T+N0) that derives significant survival benefit from adjuvant immunotherapy, while suggesting the lack of efficacy in others (T+N+, T0N+). By enabling pathology-driven, personalized adjuvant therapy decisions, this stratification represents a significant step forward in the precision management of esophageal cancer.

## Data Availability

The original contributions presented in the study are included in the article/supplementary material. Further inquiries can be directed to the corresponding authors.
